# Soluble Chenopodin–Alginate and Chenopodin–Chitosan Nanocomplexes as Building Blocks for Food Emulsion Gels

**DOI:** 10.3390/gels12080699

**Published:** 2026-08-05

**Authors:** Tatiana Isabel Romo, Gonzalo G. Palazolo, Jorge R. Wagner, Lilian Abugoch, Cristian Tapia

**Affiliations:** 1 Departamento de Ciencia de Alimentos y Tecnología Química, Facultad de Ciencias Químicas y Farmacéuticas, Universidad de Chile, Santiago 8380494, Chile; tatianaisabelromoortiz@gmail.com; 2 Facultad de Ciencias de la Salud, Universidad Tecnológica Empresarial de Guayaquil (UTEG), Guayaquil 090616, Ecuador; 3 Laboratorio de Investigación en Funcionalidad y Tecnología de Alimentos, Departamento de Ciencia y Tecnología, Universidad Nacional de Quilmes, Buenos Aires B1876BXD, Argentina

**Keywords:** protein–polysaccharide complexes, chenopodins, sodium alginate, chitosan, emulsions, stability, dressings

## Abstract

This study evaluates the network-forming and gelation capabilities of quinoa protein (QP) nanocomplexes formed with alginate (QP–Al) and chitosan (QP–C) for the development of structured food emulsion gels and their application in reduced-fat food dressings. Rheological and nanometric characterisation revealed that QP–C complexes exhibited strong shear-thinning behaviour and particle-size instability with increasing concentration, indicating the breakdown of an organised internal network at rest. Conversely, QP–Al showed Newtonian behaviour, smaller particle sizes (~100–250 nm) and high surface charge stability. Upon oil incorporation, the chitosan-based systems underwent an abrupt, concentration-dependent transition from a liquid-like state to a solid-like gel network between 1.2% and 1.6% w/v chitosan. The EQP–C8 gel network exhibited severe structural fragility, degrading into a purely viscous fluid over 28 days. Conversely, the alginate-based system (EQP–AL8) formed a weak physical hydrogel network characterised by a progressive build-up of structure that resisted creaming and maintained structural integrity across temperature changes. EQP–AL8 was successfully used to develop a plant-based, reduced-fat dressing with high organoleptic acceptance; 98% of participants were willing to purchase the product. These findings demonstrate that QP–AL8 provides a clean-label technological path to designing tunable, highly stable food emulsion gels.

## 1. Introduction

In recent years, the food industry has witnessed a significant shift towards plant-based proteins as a sustainable and healthy alternative to animal-derived ingredients [1,2,3]. Quinoa protein (QP) is an effective emulsifier, primarily owing to its 11S globulin (chenopodin) and 2S albumin fractions. QP is a high-quality, complete protein [4], containing a balanced profile of all essential amino acids, particularly lysine and methionine, which are often limiting in other cereals. QP stabilises oil-in-water emulsions through 11S globulin (chenopodin), which binds quickly to the oil–water interface through *β*-sheet conformations. It also involves 2S albumin, which stabilises droplets through a hydrophobic core rich in *α*-helices [5]. Despite their potential, the performance of QP-stabilised emulsions is highly sensitive to environmental conditions, particularly pH and ionic strength. Near the isoelectric point ([pI] 4.5), protein solubility and surface activity decrease substantially, yielding droplet coalescence. Furthermore, high electrolyte concentrations can screen electrostatic charges, reducing the zeta potential (*ζ*) and compromising the physical stability of the system [6]. Therefore, the interfacial architecture of oil droplets can be modified by incorporating polysaccharides. These biopolymers form thick, charged layers that enhance stability through combined steric and electrostatic repulsion [7,8,9]. Notably, the formation of soluble complexes between chenopodins (QPs) and polysaccharides, such as the anionic sodium alginate (Al) and cationic chitosan (C), above the pI of the protein, can significantly alter protein functionality. The primary driving force for QP–chitosan (QP–C) was electrostatic interaction, whereas QP–alginate (QP–Alg) was stabilised by combining hydrophobic interactions, hydrogen bonding and weak electrostatic forces. Circular dichroism analysis denoted a decrease in α-helix content and an increase in β-sheets, suggesting re-organisation of the protein structure upon binding. These complexes increased the thermal stability of the QP, enhancing its heat resistance in food applications [10]. 

The rheological and microstructural effects of gums (guar, locust bean and xanthan gums [XG]) on quinoa protein isolate (QPI) gels have been explored. For XG at pH > pI, where QPI has a negative charge, the electrostatic attraction can occur between the anionic gum (XG) and the cationic amino acids located in some protein patches of QPI. This interaction results in more compact, denser structures and greater gel strength at a higher XG concentration (0.2%) [11]. 

The use of soluble QP–C and QP–AL nanocomplexes for the design of emulsion gels or structured continuous-phase hydrogels remains unexplored. Understanding how the specific QP/polysaccharide ratio influences viscoelastic networks and gel mechanics is crucial for building functional food emulsions. Therefore, this study evaluated the potential of soluble nanocomplexes (QP/AL and QP/C) to form structural hydrogel networks that encapsulate reduced-fat oil-in-water (o/w) emulsions. The effects of biopolymer ratios on viscoelastic properties, gelation thresholds and microstructural network density were systematically investigated under varying thermal conditions. These structural features were then correlated with long-term physical stability and sensory performance.

## 2. Results and Discussion

### 2.1. Flow Characterisations of Continuous Phases Based on QP–AL and QP–C Nano-Complexes

**Figure 1:**
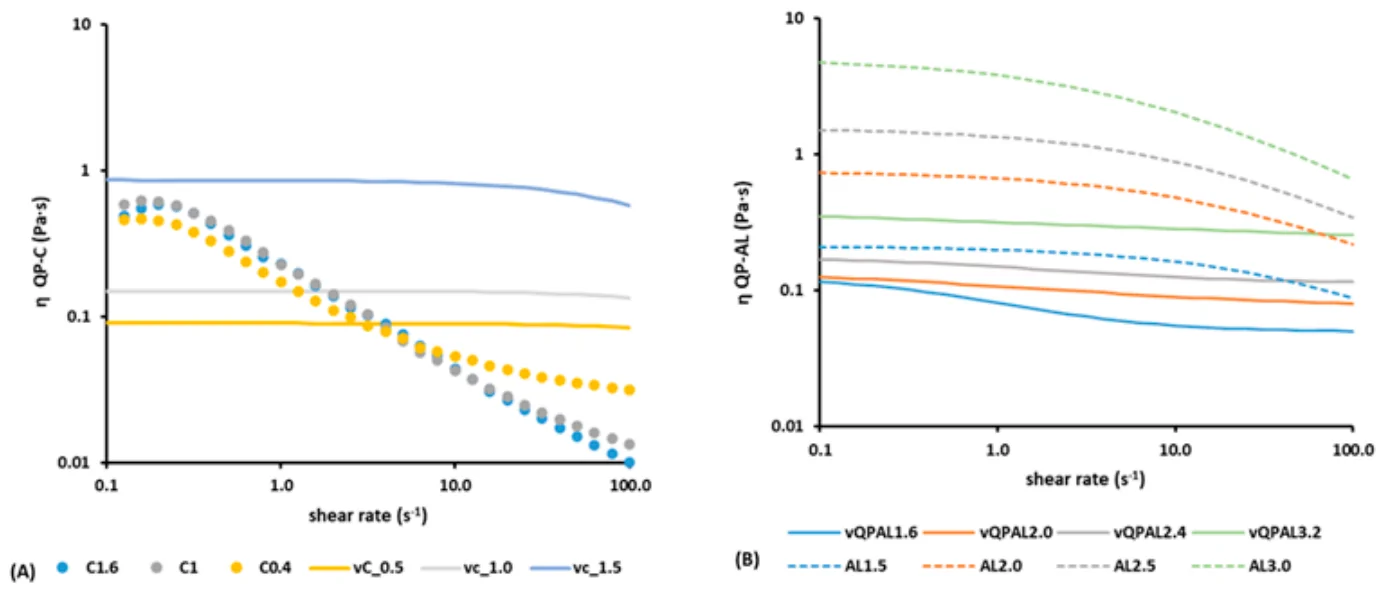
Rheological behaviour fitted to the Cross model of (**A**) QP-C (C % w/v) nanocomplexes compared with chitosan solutions (vC % w/v) and (**B**) QP-AL (vQP-AL % w/v) nanocomplexes compared with alginate solutions (Al % w/v).

The network-forming capability of the quinoa protein–polysaccharide complexes was first evaluated in their continuous phases in order to understand their macromolecular conformation before oil addition. Figure 1A shows the rheological behaviour of QP–C nanocomplexes (C 0.4%, 1.0% and 1.6% w/v) compared with chitosan solutions (C 0.5%, 10% and 1.5% w/v) at comparable concentrations obtained from the parameters fitted to the Cross model [12] as presented in Table 1. QP–C exhibits strong shear-thinning behaviour. Its viscosity is relatively high at low shear rates (approximately 0.4–0.6 Pa·s) and decreases rapidly as the shear rate increases, reaching approximately 0.01–0.03 Pa·s at 100 s^−1^. This sharp decline suggests an organized internal network or an entangled pre-gel polymer structure at rest, which undergoes severe structural breakdown or chain alignment under the influence of external flow fields. In contrast, chitosan solutions at similar concentrations exhibit a stable Newtonian or near-Newtonian behaviour. The QP–C nanocomplex contains low-molecular-weight chitosan with an average molar mass of 269 kDa and a 21.7% DA obtained from Sigma-Aldrich Inc. (St. Louis, MO, USA). Calero et al. [12] used a chitosan with similar properties (Mw: 190–310 kDa and DA (15.0–25.0%) from the same supplier, Sigma-Aldrich Inc.). The intermediate molecular weight of this chitosan (269 kDa) favours chain entanglement and steric bridging at critical concentrations, directly driving the strong shear-thinning behaviour and influencing the subsequent emulsion gelation threshold. Figure 1B shows the rheological behaviour of the QP–AL nanocomplexes (vQP–AL at concentrations of 1.6%, 2.0%, 2.4% and 3.2% w/v), fitted to the Cross model as described in Section 4.4. This has been compared with alginate solutions (Al at concentrations of 1.5%, 2.0%, 2.5% and 3.0% w/v) at similar concentrations obtained from the literature [13]. Alginate solutions exhibit strong shear-thinning behaviour and a pronounced concentration dependence. Although QP–AL nanocomplexes exhibit weak shear-thinning behaviour, their viscosity declines slightly at higher shear rates, making them closer to Newtonian behaviour. Furthermore, QP–AL nanocomplexes exhibit significantly lower viscosity at low shear rates than the alginate solutions. The QP–AL nanocomplex was prepared using a low-viscosity alginic acid sodium salt from M. pyrifera with an average molar mass of 162 kDa and an M/G ratio of 1.2 (Sigma-Aldrich Inc.). Mancini et al. [13] evaluated pristine sodium alginate from the identical botanical source (*M. pyrifera*) with a comparable molecular mass (250.5 kDa), showing typical non-Newtonian shear-thinning behaviour that serves as an appropriate baseline to contrast the lower viscosity and modified flow properties observed in our QP–AL nanocomplexes. On the other hand, the relatively low molecular weight of the sodium alginate (162 kDa) prevents excessive chain entanglement, keeping the continuous-phase viscosity low while providing sufficient electrostatic and steric shielding to stabilize the smaller nanocomplexes.

**Figure 2:**
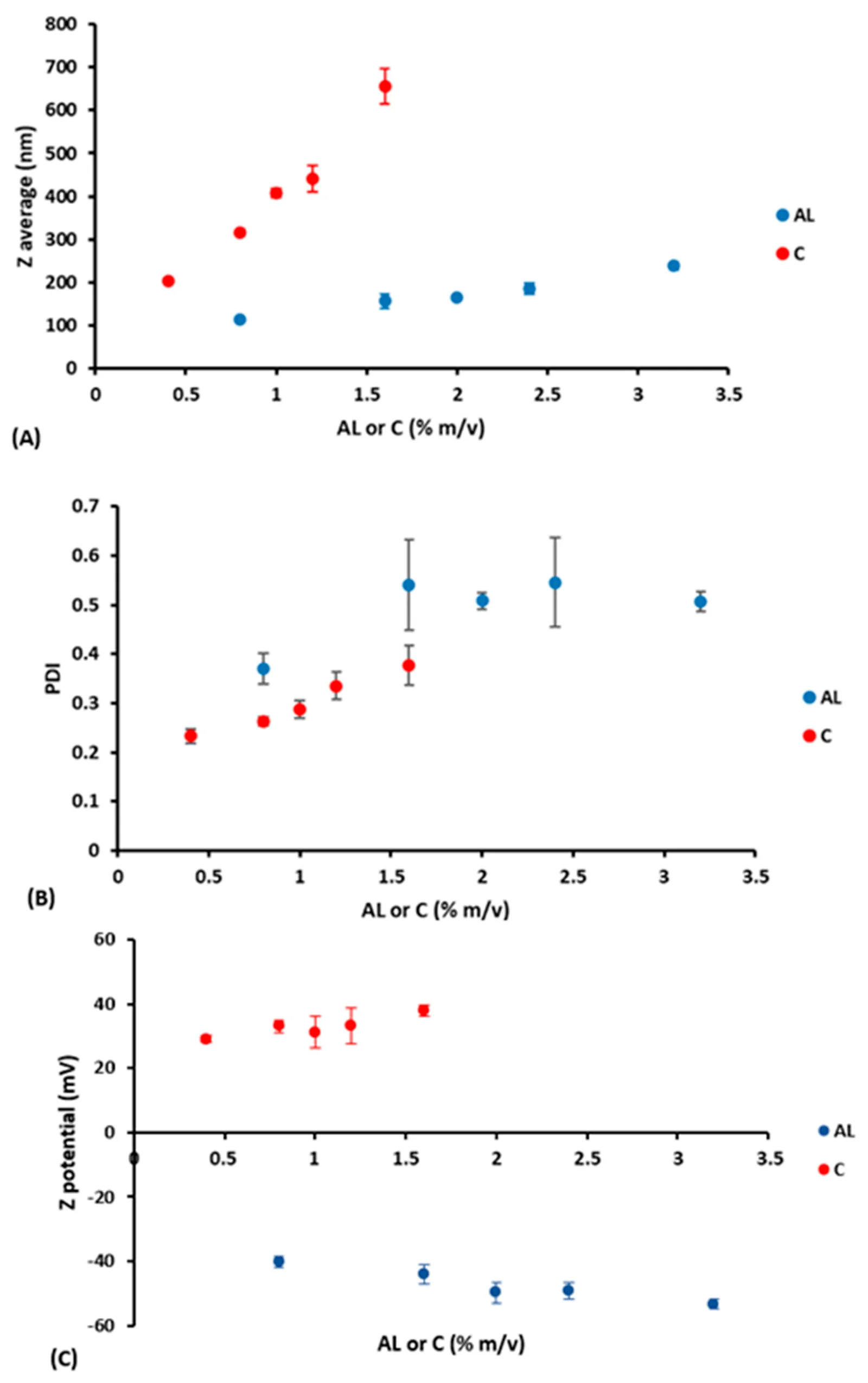
Nanometric measurements: (**A**) Z-average, (**B**) PDI and (**C**) Z-potential of QP-AL and QP-C nanocomplexes [10].

Figure 2A–C show the nanometric measurements (Z-average, PDI and Z-potential) for the QP–AL and QP–C nanocomplexes [10]. Comparing the size (Z-average, and uniformity [PDI]) and surface charge (Z-potential) indicated that QP–AL has a smaller particle size (~100–250 nm) and remains stable when the concentration increases. Although QP–C has a large particle size (~200–650 nm) that increases rapidly with concentration, it is more monodisperse (0.2–0.4) and exhibits narrower distribution than QP–AL, which is less uniform (0.35–0.55) and exhibits a broad distribution. Both nanosystems maintain Z-potential magnitudes (>30 mV), indicating excellent resistance to aggregation. In conclusion, QP–AL is the more stable nanosystem. Although QP–C produces more uniform particles, it rapidly loses stability because the particle size increases with concentration. QP–AL shows greater size variation; however, the particles have a strong electric charge that keeps them separated and maintains their size reliably across concentrations. 

Both nanosystems maintain Z-potential magnitudes (>30 mV), indicating excellent resistance to aggregation. In conclusion, QP–AL is the more stable nanosystem. Although QP–C produces more uniform particles, it rapidly loses stability with increasing material (particle size increases with concentration). QP–AL shows greater size variation; however, the particles have a strong electric charge that keeps them separated and maintains their size reliably across concentrations. Although both systems show high zeta potential (>30 mVolt), this parameter only reflects initial electrostatic repulsion. The lower stability of QP–C stems from its electrostatic nature, which is prone to charge-screening and bridging flocculation over time. Conversely, QP–AL is stabilized by cooperative hydrophobic forces and hydrogen bonds, providing robust steric shielding and a more stable hydrogel matrix.

### 2.2. Characterisation of the Quinoa Protein-Alginate (QP–AL) and Quinoa Protein-Chitosan (QP-C) Emulsions-Gel Matrices

#### 2.2.1. Flow Disruption and Viscosity Transitions of Emulsion Gel Networks of QP–AL and QP–CH

**Figure 3:**
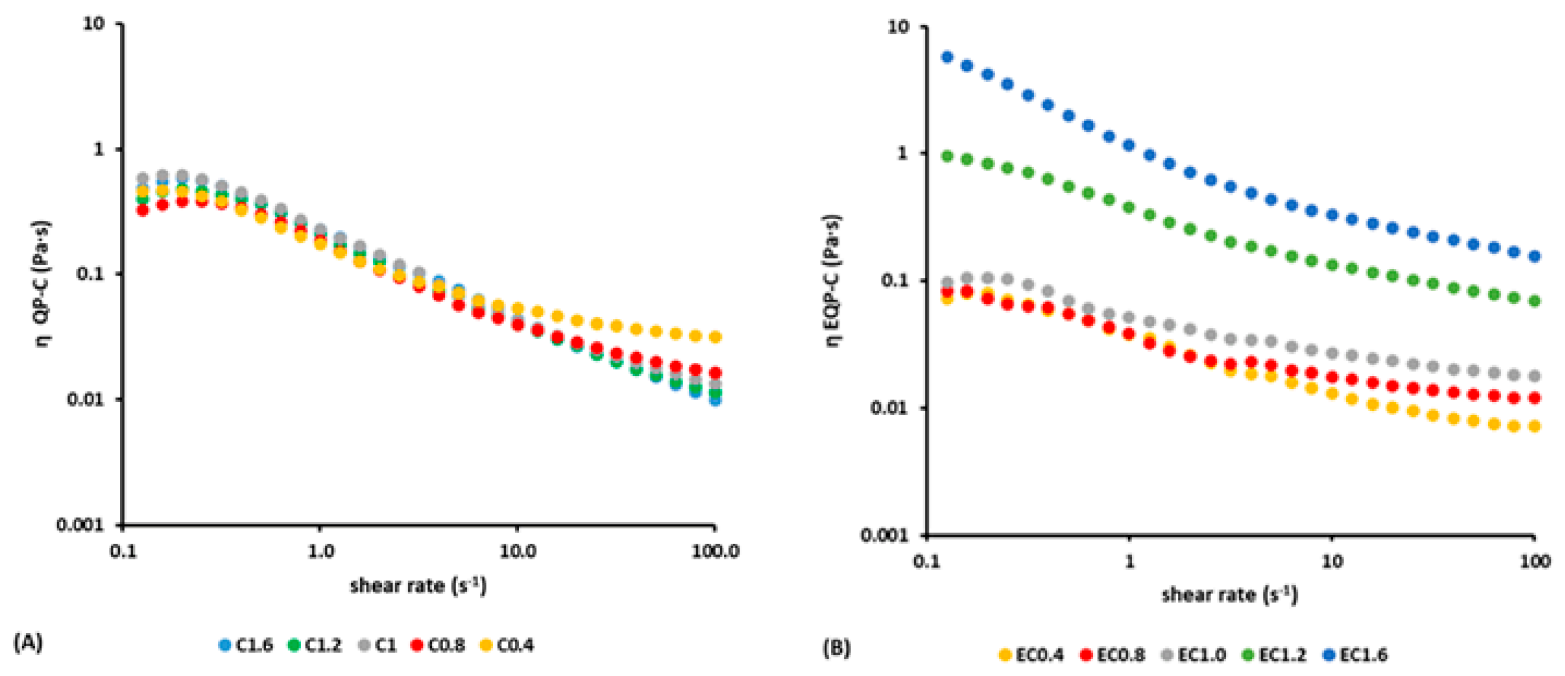
Flow characterisations of (**A**) QP-C nanocomplexes and (**B**) EQP-C emulsion gels made with sunflower oil in an o/w ratio = 1:2.

As can be seen in Figure 3A, QP–C nanocomplexes exhibit significantly lower viscosity values and a more tightly clustered distribution. The maximum observed viscosity (at low shear rates) is less than 1 Pa·s. When oil droplets are introduced, a tight structural framework forms, making the EQP–C systems significantly more viscous than their respective continuous phases. Accordingly, Figure 3B, EQP–C shows that emulsions exhibit a far wider and typically higher range of viscosities. For instance, the sample EC1.6 reaches a viscosity of approximately 6–7 Pa·s at low shear rates. Notably, EQP–C shows a clear dependence on chitosan concentration across the entire shear-rate range, whereas QP–C exhibits low sensitivity to chitosan concentration; all curves (C1.6–C0.4) are clustered within the same order of magnitude (0.1–1.0 Pa·s at low shear). Specifically, the QP–C nanocomplexes exhibit classic behaviour for polymer solutions. Region 1 (low shear) shows a distinct Newtonian plateau where the viscosity is constant (*η*_0_), and Region 2 (high shear) presents a shear-thinning region where viscosity drops linearly on the log–log scale. In contrast, the behaviour of the EQP–C emulsion gel changes drastically with concentration. At low concentrations (EC0.4–EC1.0), it resembles QP–C, whereas at high concentrations (EC1.2–EC1.6), these samples exhibit a straight line with a negative slope over nearly the entire measured range. The Newtonian plateau is likely to occur at a shear rate far lower than that measured (<0.1 s^−1^), indicating the formation of a highly structured gel network with a true yield stress.

**Figure 4:**
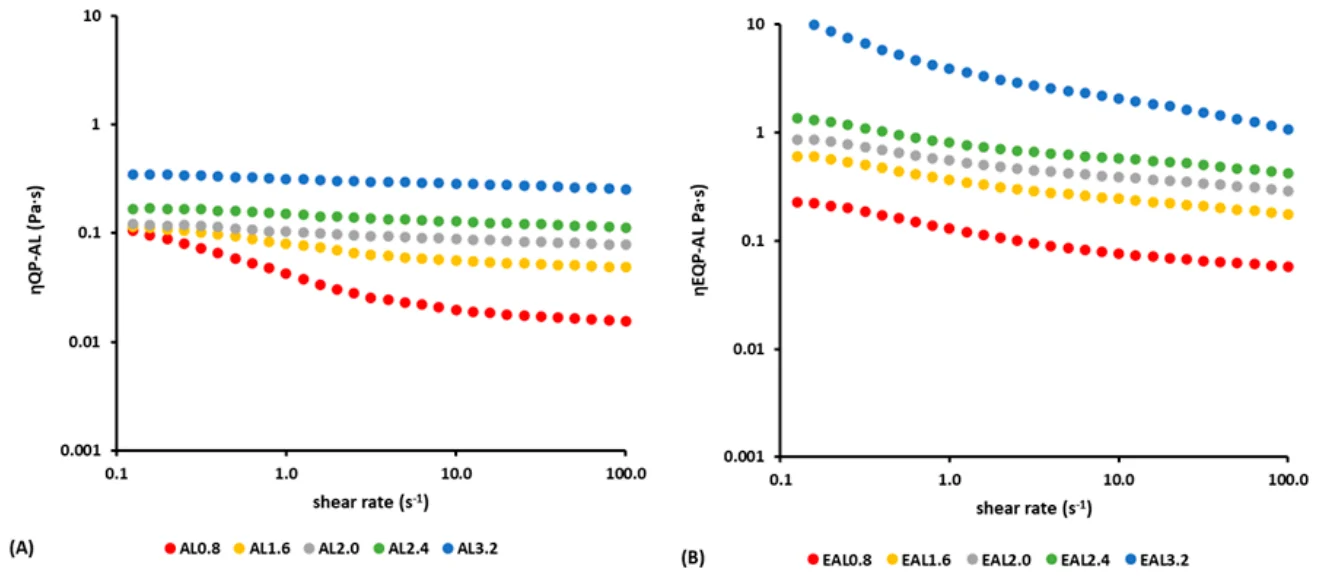
Flow characterisations of (**A**) QP-AL nanocomplexes and (**B**) EQP-AL emulsion gels made with sunflower oil in an o/w ratio = 1:2.

The viscosity values of the QP–Al nanocomplexes are low (see Figure 4A). Even at the highest concentration (AL 3.2), the viscosity remains <0.4 Pa·s. Instead, for the emulsions (EQP–Al), the viscosity values are significantly higher (Figure 4B). The highest concentration (EQP–Al 3.2) starts at approximately 10 Pa·s, which is more than an order of magnitude higher than the corresponding QP–Al sample. Most QP–AL samples (specifically AL 1.6, AL 2.0, AL 2.4 and AL 3.2) exhibit nearly Newtonian behaviour. The only exception is the lowest concentration (AL 0.8), which exhibits shear-thinning behaviour (i.e., viscosity decreases as the shear rate increases). All EQP–AL samples exhibit shear-thinning (pseudoplastic) behaviour. As the shear rate increases, viscosity decreases significantly across all concentrations. The viscosity of both materials increases as the alginate concentration increases from 0.8% to 3.2% w/v. For QP–AL nanocomplexes, the steps between concentrations are relatively uniform and small; for EQP–AL emulsions, the spacing between the curves is wider, especially at the highest level (EAL 3.2). This suggests that the modification of or increase in the concentration of EQP–AL has a more significant impact on the internal structure and resistance to flow of the material. This confirms that oil entrapment within the EQP–AL network dramatically impacts the internal structure, increasing the material’s flow resistance and producing a structural hydrogel body that breaks down cleanly under shear. From a practical standpoint, the pronounced shear-thinning behaviour of these systems offers clear processing advantages. During high-shear operations such as pumping, mixing, or volumetric filling, the rapid drop in apparent viscosity minimizes flow resistance and energy consumption. Once at rest, the recovery of high viscosity structurally stabilizes the matrix, spatially immobilizing the oil droplets and preventing gravity-driven creaming during storage.

#### 2.2.2. Determination of Dynamic Viscoelastic Behaviour of QP–Alg and QP–C Emulsions

**Figure 5:**
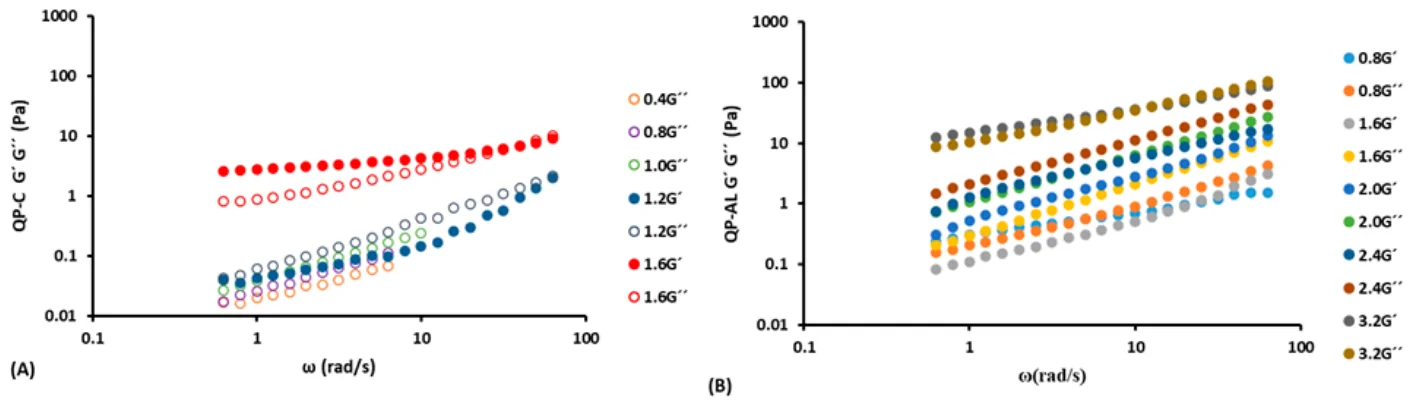
Characterisation of the dynamic viscoelastic behaviour of (**A**) EQP-C emulsion gels and (**B**) EQP-Al emulsion gels made with sunflower oil in an o/w ratio = 1:2.

As shown in Figure 5A, the EQP–C emulsions sharply transition from a liquid-like to a solid-like gel state between chitosan concentrations of 1.2% and 1.6% w/v. This abrupt transition reflects a critical percolation threshold between 1.2% and 1.6% w/v. At 1.6%, the highly entangled cationic chitosan chains bridge the negatively charged quinoa proteins at the interface. This electrostatic bridging and cross-linking of the oil droplets trigger sudden structural arrest, resulting in the formation of a three-dimensional gel network. Below 1.2% the moduli are extremely low (<1 Pa), and for the lowest concentrations (0.4, 0.8), only G″ is significant enough to be plotted; this indicates a low-viscosity liquid behaviour. At a concentration of 1.6, the storage modulus (G′, solid circles) is consistently higher than the loss modulus (G″, open circles). The dominance of G′ over G″ confirms that a gel structure has formed. Conversely, in Figure 5B, the EQP–AL emulsions show a progressive build-up of structure without a sharp transition. For most concentrations, the storage and loss moduli are almost equal. All curves, even at the highest concentration (3.2), show a steep slope. Both moduli increase significantly with increasing frequency. This behaviour is typical of entangled polymer solutions or weak hydrogel networks, which behave similarly to solids at high *ω* but flow similarly to liquids at low *ω*.

### 2.3. Creaming Rate of QP–C and QP–AL o/w Emulsions

**Figure 6:**
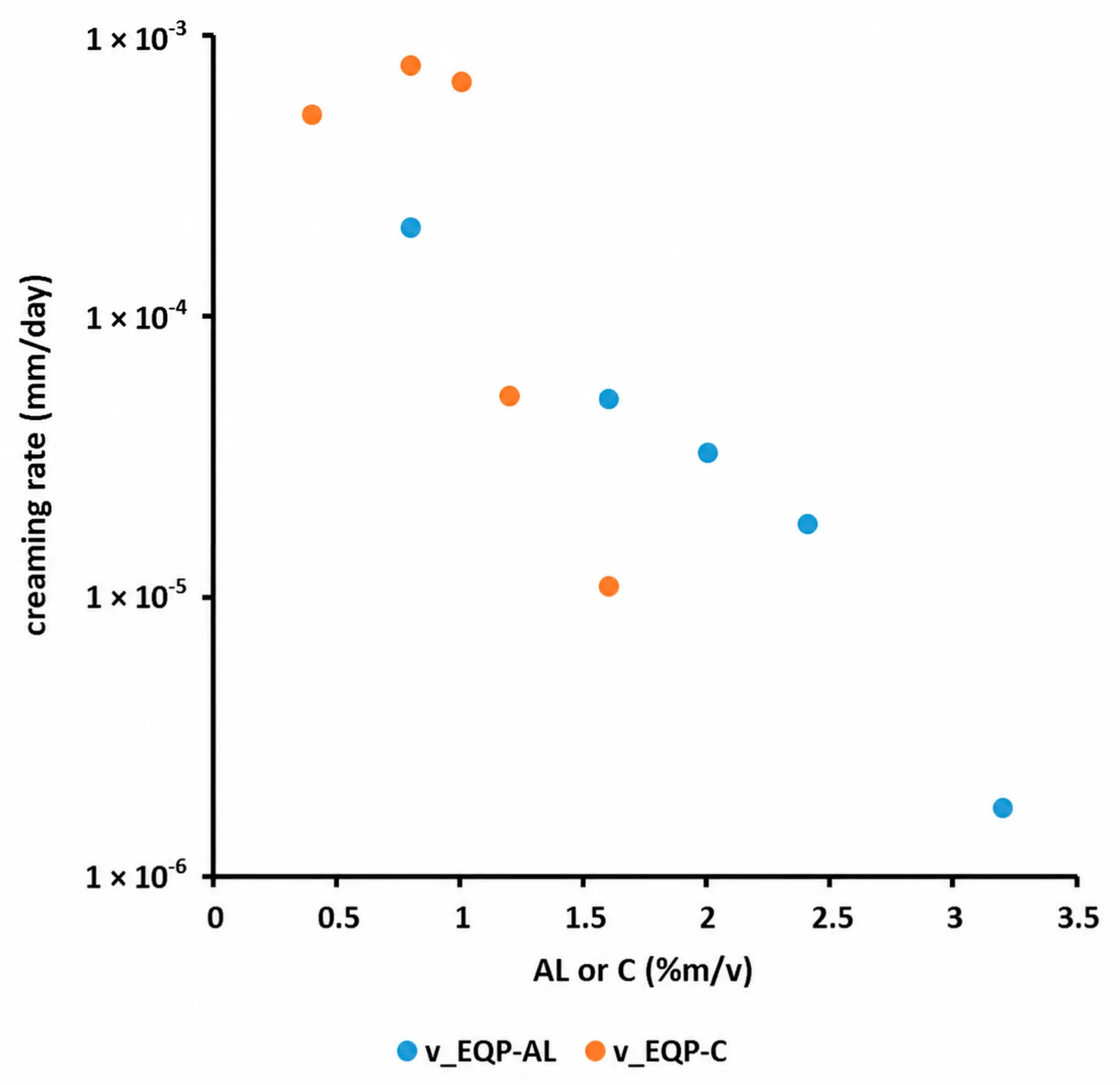
Creaming rate of EQP-C and EQP-AL o/w emulsion gels. EQP–C8 and EQP–AL8 emulsion gels contained 1.6% w/v of chitosan and 2% w/v of alginate, respectively.

The creaming rate, measured in mm/day, of QP–C and QP–AL o/w emulsions at different chitosan and alginate concentrations was estimated as described in Section 4.6 and Figure 6. The macroscale physical stability of these systems against phase separation (creaming rate, mm/day) is directly driven by the mechanical strength of their internal polymer networks. The creaming rate of loosely structured EQP–C networks is higher: at lower concentrations (<1.5% w/v), the data points denote significantly higher creaming rates than those for EQP–AL. The highest creaming rate was observed for EQP–C (approximately 1.0 × 10^−3^ mm/day), whereas the lowest was recorded for EQP–AL (approximately 1.5 × 10^−6^) at the highest concentration. EQP–C shows an extremely sharp decline. EQP–C starts high but drops precipitously between concentrations of approximately 1.0% and 1.6%. The rate decreases by nearly two orders of magnitude within a short increase in concentration. EQP–AL decreases more gradually. EQP–AL follows a steadier, approximately linear decline on the semi-log scale. The EQP–AL system produced the most stable emulsion because it achieved the lowest overall creaming rate and maintained greater stability across most of the concentration ranges. Since the lowest creaming rates were observed at the highest concentrations of chitosan and alginate, stability studies were conducted for these two formulations.

### 2.4. Stability Studies of EQP–C8 and EQP–AL8 Emulsions 

#### 2.4.1. Stability Studies at 25 °C

**Figure 7:**
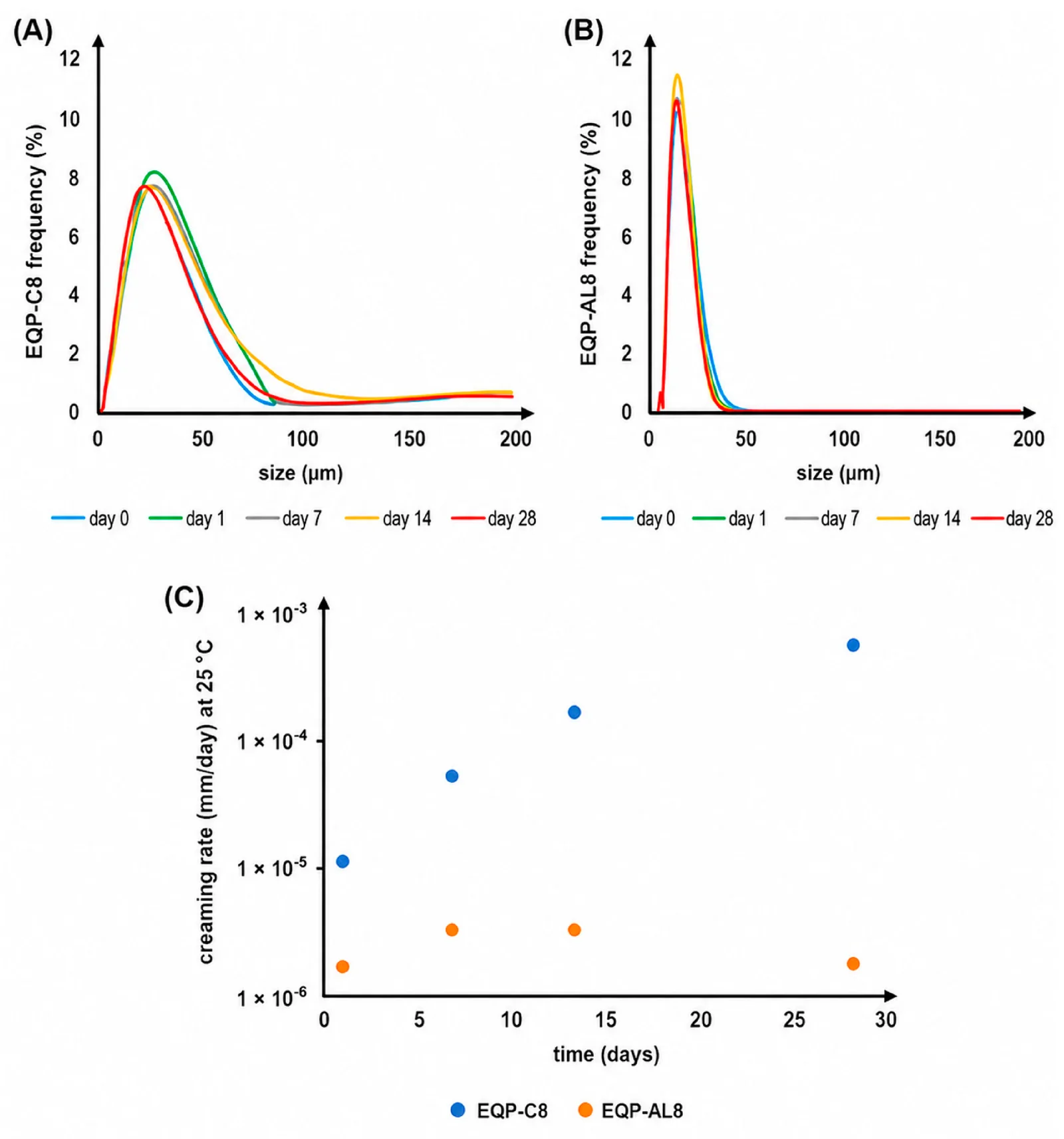
Droplet size frequency distribution (**A**,**B**) and creaming rates (**C**) of EQP-C8 and EQP-AL8 emulsions at 0, 1, 7, 14 and 28 days (25 °C).

The evolution of droplet sizes [D 3, 2] and macroscale kinetics was tracked over 28 days to monitor gel network rearrangements. The accumulated frequency of the particle-size distribution measured as [D 3, 2] of EQP–C8 and EQP–AL8 over 28 days (0, 1, 7, 14 and 28) at 25 °C is shown in Figure 7A and Figure 7B, respectively. The evolution of the creaming rate over the period was calculated per the procedure described in Section 4.6 (see Figure 7C). Figure 7A shows a broader EQP–C8 distribution, with the main body extending from 0 µm to approximately 70 µm. There is a visible difference between the earlier (blue/green lines) and later (yellow/red lines) days. In the later days (days 14 and 28), a ‘tail’ appears on the right side of the graph (from 100 to 200 µm), suggesting instability. This progressive shift toward larger sizes likely reflects a combined contribution of irreversible coalescence and bridging flocculation. Because chitosan is a highly charged cationic biopolymer, it can induce droplet clustering via electrostatic bridging, creating stable flocs that register as larger particle populations during light scattering analysis. By contrast, Figure 7B shows an extremely narrow distribution of EQP–AL8. The particles are tightly clustered within a narrow size range, approximately 0–30 µm, indicating an extremely uniform emulsion with similarly sized droplets. The curves for days 0–28 overlap almost perfectly. EQP–AL8 creates a finer, more uniform and highly stable emulsion with small particles. Figure 7C shows that EQP–C8 has significantly higher creaming rates at all time points than EQ–AL8. By day 28, the rate is nearly two orders of magnitude higher than that of the EQP–AL8 system (approaching 4.0 × 10^−4^ vs. 2.0 × 10^−6^ mm/day). This structural change signals a loss of network entrapment capacity, allowing oil droplet coalescence and a rapid increase in creaming rate. EQP–AL8 is the most stable emulsion. These differences in stability are driven by their distinct stabilising forces. While QP–C relies solely on electrostatic interactions to form a fragile network that is susceptible to time-dependent relaxation, QP–AL is supported by a cooperative framework of hydrophobic interactions, hydrogen bonds and weak electrostatic forces. This multi-interaction network provides structural flexibility, enabling polymer rearrangement (ageing) whilst ensuring the droplets remain immobilised. The physical stability of the emulsions was evaluated over 28 days of storage. The kinetics of the creaming index along with visual observation of phase separation in calibrated tubes are shown in Figure S1. Additionally, light backscattering measurements (Turbiscan) confirmed the stability behavior of QP–AL 1:4 and QP–C 1:4 formulations (Figure S2).

#### 2.4.2. Stability Studies at Different Temperatures

**Figure 8:**
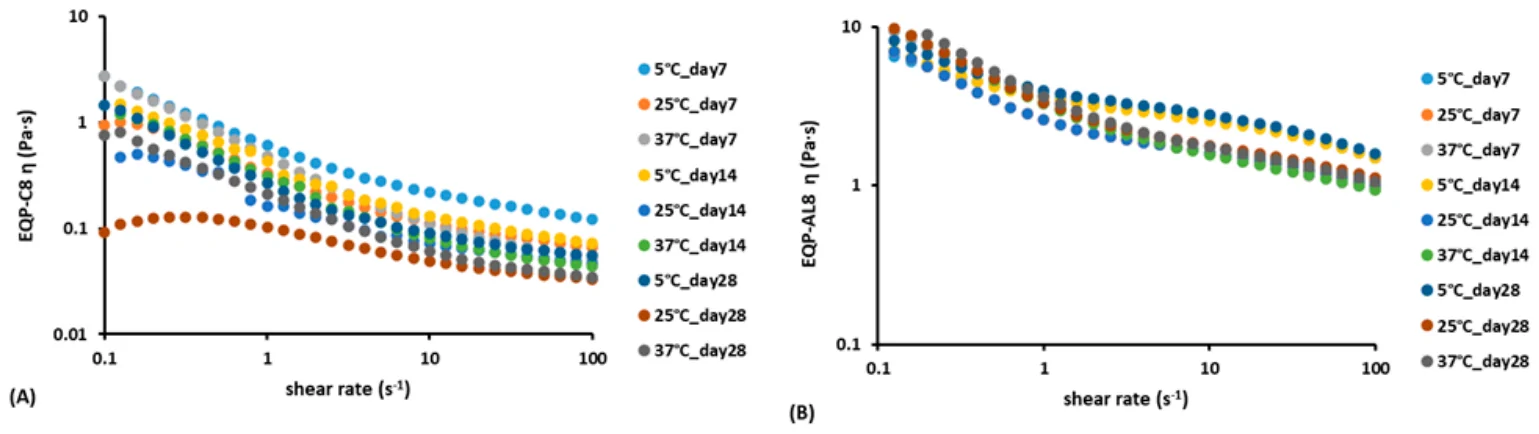
Effect of three different temperatures (5, 25 and 37 °C) on the flow behaviour of (**A**) EQP-C8 and (**B**) EQP-AL8 at days 7, 14 and 28.

Figure 8 demonstrates the effect of three different temperatures (5 °C, 25 °C and 37 °C) on the flow behaviour of (A) EQP–C8 and (B) EQP–AL8 on days 7, 14 and 28. As mentioned above, both systems exhibit shear-thinning behaviour. EQP–AL8 and EQP–C8 range from approximately 1 to 10 Pa·s and 0.04 to 2 Pa·s, respectively, across the tested shear rates. The curves for EQP–AL8 on days 7, 14 and 28 are tightly clustered, indicating high stability. The temperature has minimal impact on the flow behaviour. The curves for 5 °C, 25 °C and 37 °C substantially overlap. Instead, the viscosity of EQP–C8 decreases significantly with time at a given temperature, showing clearer stratification by temperature. At 5 °C, EQP–AL8 generally maintains a higher viscosity and represents a far more stable formulation, consistently exhibiting elevated viscosity regardless of storage time or temperature within the tested range. By contrast, EQP–C8 is thinner and highly unstable, showing significant viscosity loss over time, a process accelerated by higher temperatures.

**Figure 9:**
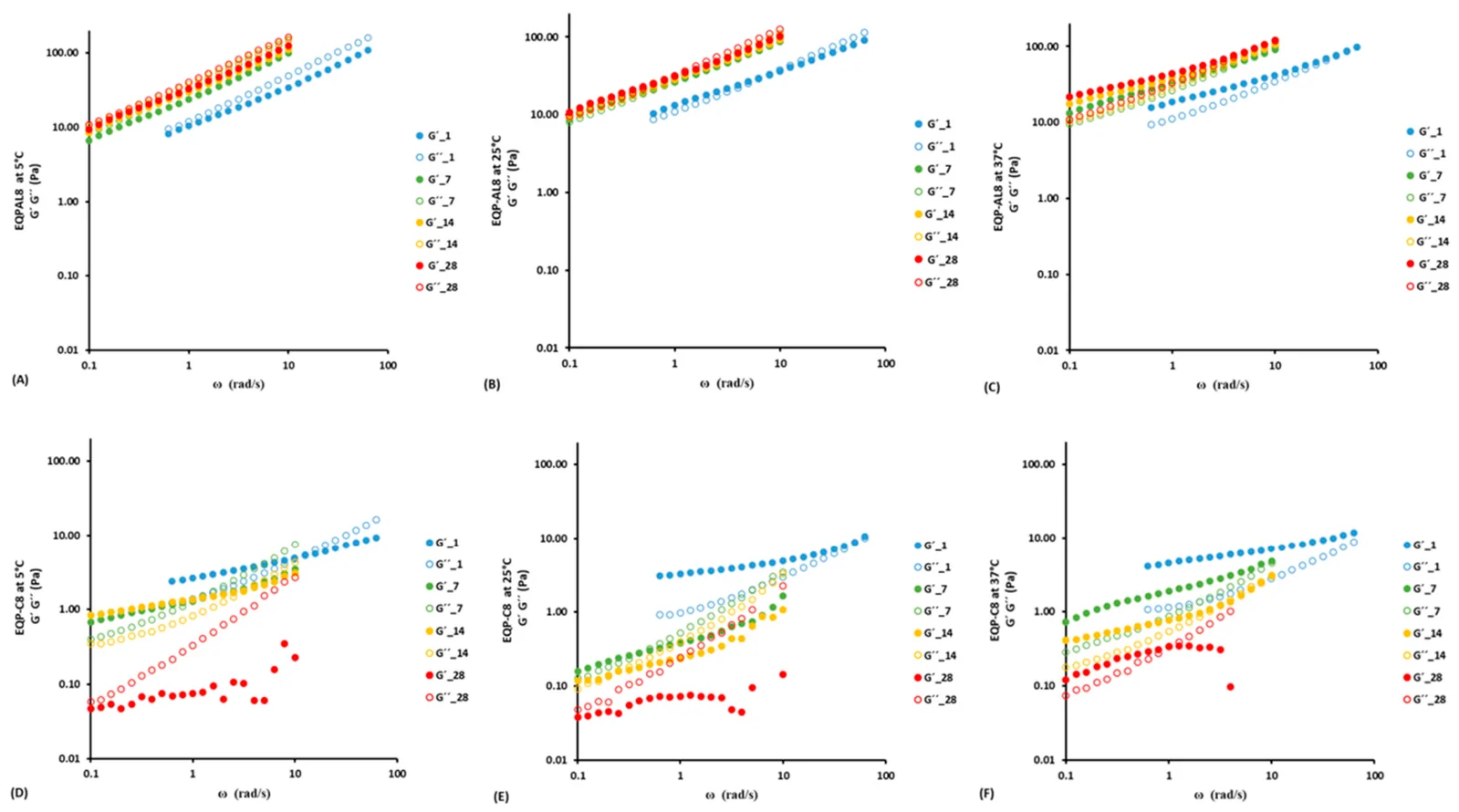
Effect on the viscoelastic behaviour of EQP-AL8 and EQP-C8 emulsion gels at days 1, 14, and 28 at three temperatures: (**A**) 5 °C, (**B**) 25 °C, and (**C**) 37 °C for EQP-AL8 and (**D**) 5 °C, (**E**) 25 °C and (**F**) 37 °C for EQP-C8.

Figure 9 shows the effect on the viscoelastic behaviour of EQP–AL8 and EQP–C8 emulsions on days 1, 7, 14 and 28 at three temperatures: (A) 5 °C, (B) 25 °C and (C) 37 °C for EQP–AL8 and (D) 5 °C, (E) 25 °C and (F) 37 °C for EQP–C8. For the emulsion gel based on EQ–AL8 at 5 °C, the predominant trend is a clear ageing or structuring effect: as time progresses from days 1 to 28, the material becomes significantly stiffer and more structured, as indicated by the upward shift in both moduli. The greatest change occurs during the 1st week (days 1–7), followed by gradual hardening through day 28. 

Across all 28 days, EQP–AL8 maintains a viscous behaviour; the loss modulus (G″) remains slightly higher than the storage modulus (G′). At 25 °C, the EQP–AL8 emulsion maintains the same behaviour as at 5 °C. At 37 °C, a clear change is noted in the viscoelastic behaviour. There is a solid-like behaviour for each day of study, with G′ > G″. Both moduli increase with angular frequency, a typical behaviour for viscoelastic hydrogels or soft solids. The emulsion stabilised by EQP–C8 is considerably less stable than that stabilised by EQP–AL8. At 5 °C, modulus values clearly decrease with increasing age from days 1 to 28, indicating that the material is becoming softer and losing its structural integrity over time. The EQP–C8 emulsion gel transitions from a stiffer, structured viscoelastic material (day 1) to a stable gel-like state (days 7–14) and ultimately breaks down into a predominantly viscous fluid with negligible elasticity by day 28. At 25 °C, EQP–C shows the same behaviour as at 5 °C. The emulsion transitions from a solid-like gel structure at day 1 to a liquid-like fluid by day 28. Storage at 25 °C degrades the EQP–C8 network. By day 7, the strong gel of day 1 has become a weak gel, readily yielding to faster deformations. By day 28, the gel structure is effectively gone, leaving a liquid. At 37 °C, on day 1, the emulsion shows the strongest gel state. G′ is consistently higher than G″ across the entire frequency range. Progressive softening occurs on days 7 and 14. On day 7, G drops to 0.8–5 Pa·s. On day 14, the degradation continues, with G′ decreasing to 0.4–3 Pa·s. Despite the drop in strength, G′ remains higher than G″, suggesting that the material physically behaves as a solid, but its internal network is breaking down. The emulsion stiffened from a stable soft gel on day 1 to a weak and extremely weak gel on days 7 and 14 and then collapsed on day 28.

#### 2.4.3. Estimation of Creaming Stability of EQP–AL8 and EQP–C8 Emulsions from Backscattering Data

**Figure 10:**
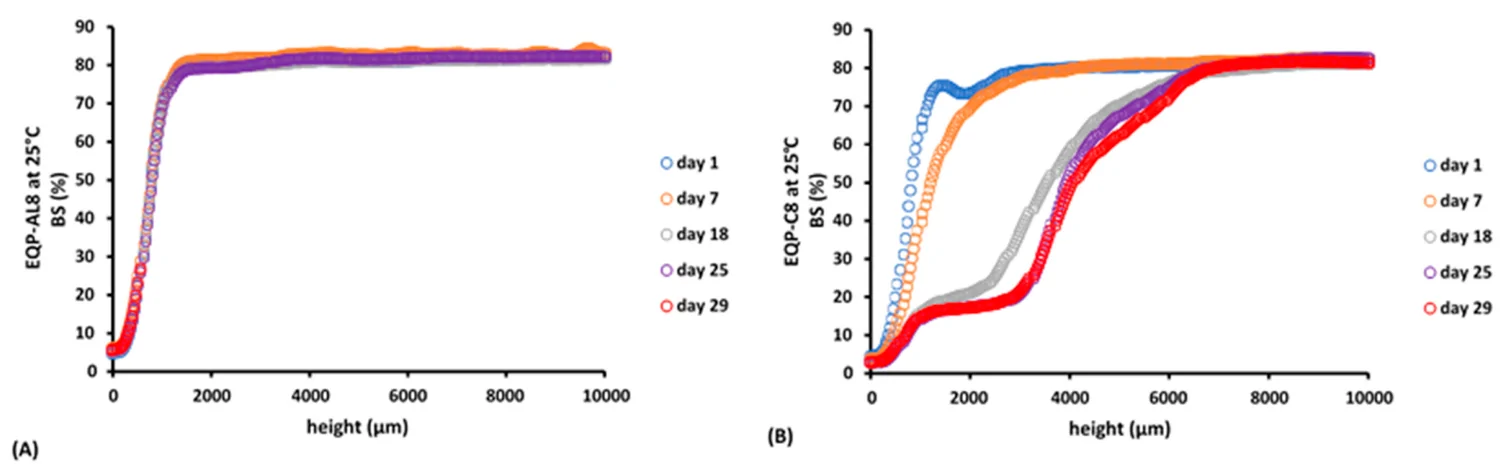
Sigmoidal shape of the backscattering emulsion data of (**A**) EQP-AL8 and (**B**) EQP-C8 emulsions between day 1 and day 29.

Figure 10 shows the sigmoidal shape of the backscattering emulsion data of EQP–AL8 and EQP–C8 between days 1 and 29. From the backscattering data obtained with TURBISCAN, the height data between 0 and 10,000 μm were fitted to the Boltzmann sigmoid described in Section 4.7. From this equation, the H50 value was obtained, representing the height of the inflexion point (the creaming front) for each day (Table 1).

**Table 1:** Backscattering profiles and determination of H50 values for EQP–AL8 and EQP–C8 emulsion gels.

Emulsion Gel	EQP–AL8			EQP–C8		
Time (Days)	Front Position (H_50_) (μm)	Change from Day 1 (μm)	Migration Velocity (μm/day)	Front Position (H_50_) (μm)	Change from Day 1 (μm)	Migration Velocity (μm/day)
1	700.5			729		
7	694.5	−6.0		840	111	
18	692.8	−7.7		3219	2490	
25	712.8	12.3		3900	3171	
29	711.4	10.9	0.4	4027	3298	117.8

The change in the front position is <15 μm, which is invisible to the naked eye. At 29 days, the migration velocity is 0.40 μm/day. By contrast, EQP–C8 emulsions are unstable. The interface is moving upwards significantly over time. The movement accelerates markedly between days 7 and 18, suggesting a coalescence or flocculation event that accelerates separation. At 29 days, the migration velocity is 117.8 μm/day.

**Figure 11:**
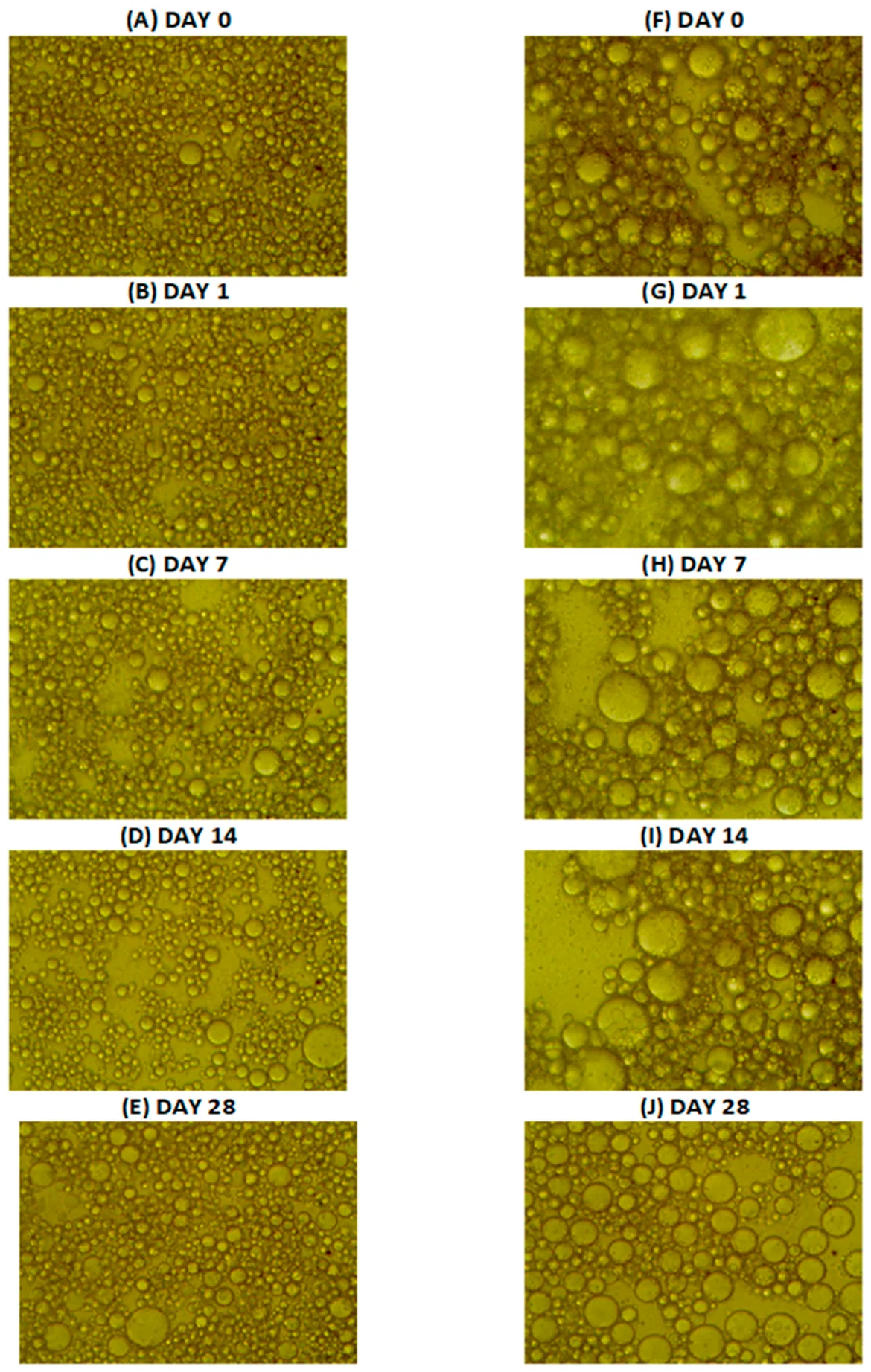
Microscopic images of (**A**–**E**) EQP-AL8 and (**F**–**J**) EQP-C8 emulsion gels stored for 28 days at 25 °C.

This macroscale behaviour is confirmed by the optical microstructure images (see Figure 11). Figure 11 shows that all the emulsions exhibit spherical particles without deformation or oil release. This microstructure is typical of concentrated emulsions with a packed distribution of oil drops [14]. However, the EQP–AL8 emulsions exhibited more compact networks, with minimal inter-particle spacing, particularly during the initial storage stages. Furthermore, the EQP–AL8 emulsions had smaller particles than the QP–C emulsions, and their diameters remained constant throughout the storage time. By contrast, in EQP–C8 emulsions, the particle size increased with storage time. In addition, the EQP–AL8 emulsions showed more homogeneous particle distributions than the EQP–C8 emulsions. These findings were consistent with the frequency-accumulated particle-size distribution measured as [D 3, 2] for EQP–C8 and EQP–AL8 (Figure 7A,B). Similar to EQP–AL8 emulsions, various reduced-fat dressings presented in the literature showed no significant and small change in particle size until after 2 months and 30 days of storage, respectively [15,16]. Similar behaviour to that of EQP–C8 emulsions was observed for high-fat mayonnaises after 40 days of storage [15] and for reduced-fat dressings after 30 days [17]. The EQP–AL8 system stands out by forming a stable, cold-set weak hydrogel network. This matrix effectively immobilises droplets and prevents phase separation without requiring high-temperature processing. In summary, a direct structure–property relationship governs these systems: the progressive rheological structuring of EQP–AL8 creates a dense continuous matrix that spatially immobilises the oil droplets, preserving the fine, homogeneous microstructure observed under microscopy and preventing macroscale phase separation. Conversely, the severe rheological degradation and viscosity loss of EQP–C8 over time lead to network collapse, directly triggering the droplet clustering and growth visible in microscopic images, which ultimately drives the rapid migration of the creaming front.

### 2.5. Reduced-Fat Dressing Based on EQP–AL8 Emulsions

The reduced-fat dressing formulation was prepared using EQP–AL8 emulsions. Colourant, flavour and various natural spices were added. Figure S3 Visual appearance of (A) emulsion formulation components and (B) final food dressing application Consumers tested the formula. Table 2 presents the nutritional composition and energy content of the food dressing.

The sensory evaluation was conducted with 60 consumers. Participant demographics were as follows: men (40%) and women (60%); age distribution: 19–28 years (78.33%), 29–38 years (13.33%), 39–48 years (5%), 49–57 years (3.33%). All participants were from the Faculty of Chemical and Pharmaceutical Sciences, University of Chile.

**Figure 12:**
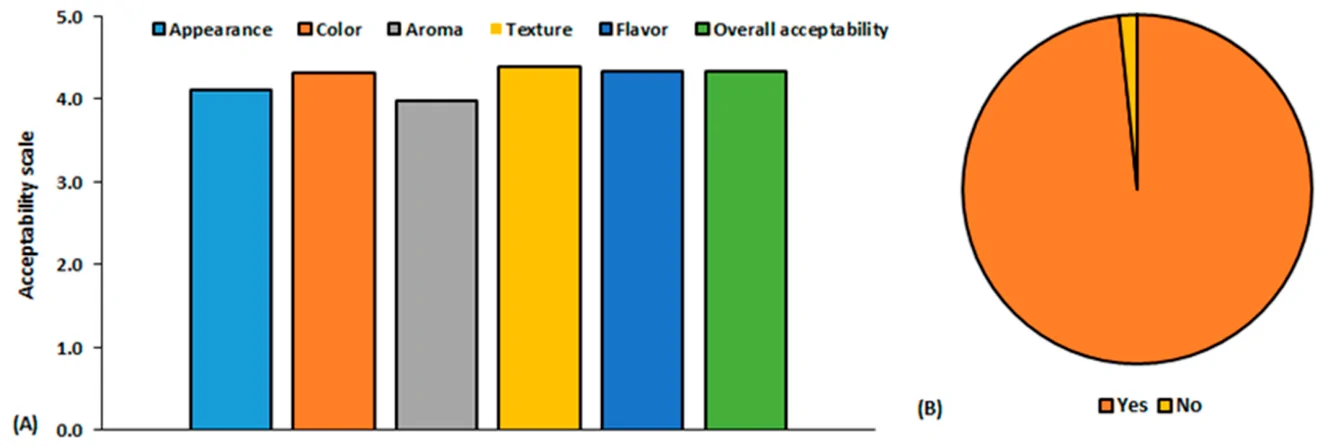
(**A**) Sensorial acceptability of the dressing and (**B**) willingness to purchase the dressing.

The main component was water in the aqueous phase of the EQP–AL8 emulsion gels at 1:4 (67%), followed by fats (28.77%). The minor components present in the sample were insoluble fibre (1.97%), carbohydrates (1.09%) and proteins (1.03%). Sunflower oil, containing polyunsaturated fatty acids (18.70%) and monounsaturated fatty acids (6.96%), was used. The dressing had neither trans fat nor cholesterol. Based on its nutritional composition, the dressing obtained had a caloric value of 26.76 kcal/suggested portion (Table 2). The flavouring and colouring used to prepare the food dressing were non-caloric; they did not contribute energy or nutrition to the food product. Moreover, Al is an insoluble dietary fibre [18] and thus does not contribute calories to the dressing. In contrast, mayonnaise and regular dressings have lipid contents of 80–90% and caloric values of 710–775 kcal/100 g of product [15,19,20,21,22,23]. The developed reduced-fat dressing formula has a low total lipid content (28.77%) and a calorie reduction of >60%. Thus, the formulation qualifies as a product classified as reduced in fat and calories under Chilean food health regulations [24]. Because the emulsion has sunflower oil, the dressing could claim to be a product free of trans fat, saturated fat and cholesterol, based on Food Sanitary Regulations [24]. The sensory evaluation of the dressing was conducted with 60 consumers. The participant distribution was 40% men and 60% women in the following age ranges: 19–28 (78.33%), 29–38 (13.33%), 39–48 (5%) and 49–57 (3.33%) years, respectively. The participants in this acceptability test were undergraduate and graduate students, academics and administrative staff from the School of Chemical and Pharmaceutical Sciences of the University of Chile. Figure 12A shows the acceptability of the food dressing across various sensory attributes, measured on a five-point hedonic scale. Figure 12B shows the consumers’ willingness to buy the dressing.

## 3. Conclusions

This research establishes that EQP–AL nanocomplexes are more effective than EQP-C for designing structured food emulsion gels. Rheological profiles demonstrate that, although the chitosan-based system (EQP-C8) exhibits an abrupt, threshold-driven gelation transition (G’ > G’’), the resulting network is structurally fragile and gradually transforms into a viscous fluid over time. This process is accelerated at 37 °C. In contrast, the alginate-based system (EQP–AL8) forms a highly robust physical hydrogel matrix. This network displays an advantageous self-assembling ageing effect at low temperatures and transitions into a firm, soft solid gel state (G’ > G’’) at elevated temperatures (37 °C). Microstructural and sigmoidal backscattering analyses suggest that the dense hydrogel matrix immobilises oil droplets and minimises the potential for coalescence. This results in zero visible phase separation over 29 days. While these macroscopic observations are consistent with the proposed network formation and structural breakdown mechanisms, further molecular-level analysis would definitively map the exact kinetics of droplet rearrangement. The result is a robust, consistent texture in the final reduced-fat dressing. With a 60% reduction in calories, this formulation improves the nutritional value of quinoa’s complete protein profile, which is rich in lysine and methionine. This offers a clean-label solution that is aligned with health and sustainability.

## 4. Materials and Methods

### 4.1. Materials

Quinoa flour (*Chenopodium quinoa* Willd) supplied by the Sociedad Comercial y Agrícola Promauka Ltd., Paredones，Chile. Low-molecular-weight chitosan with an average molar mass of 269 kDa and a 21.7% degree of acetylation (DA) and low-viscosity alginic acid sodium salt from *Macrocystis pyrifera* with an average molar mass of 162 kDa and an M/G ratio of 1.2 were purchased from Sigma-Aldrich Inc. (St Louis, MO, USA), and their properties were experimentally determined [25]. Sunflower oil was provided by Molino Cañuelas (Buenos Aires, Argentina). The materials used for the preparation of the low-fat matrix were orange-red colourant obtained from achiote seed (*Bixa orellana* L.) and flavouring powder identical to that naturally purchased from IFF Frutarom Chile S.A. (Santiago, Chile), fine iodised salt acquired from K + S Chile S.A. (I Region, Iquique, Chile), garlic powder from Good Food S.A. (Santiago, Chile) and Merquén by ICB S.A. (Santiago, Chile). All solutions and emulsions were prepared using deionised water.

### 4.2. Preparation of Quinoa Protein Isolate Enriched in Chenopodins

QPI enriched in chenopodins (QPs) was obtained by alkaline extraction and isoelectric precipitation at pH 8 and 4.5, respectively, using defatted quinoa flour as the raw material [10]. QP was neutralised, freeze-dried (model FD5508, Ilshin Biobase, Dongducheon-si, Republic of Korea) and stored at 4 °C until use. The protein content determined by the Kjeldahl method [26] was 84.8% ± 0.64%.

### 4.3. Preparation of QP–AL and QP–C Continuous Phases and o/w Emulsions

Stock solutions of 4% (w/v) Al (water), QP (water adjusted to pH 8) and 2% C (0.1 M acetic acid) were prepared. Blends of QP–AL or QP–C were prepared using the solutions mentioned above and by varying the QP–polysaccharide ratios at 1:4, 2:3, 1:1, 3:2 and 4:1. pH of QP–AL and QP–C blends was adjusted to 6 and 5.5, respectively. O/w emulsions were prepared by adding sunflower oil to the continuous phase (aqueous phase:oil ratio, 2:1) and homogenising with an Ultra-Turrax homogeniser (IKA, Model T25, Staufen, Germany) at 16,400 rpm for 3 min.

### 4.4. Rheological Characterisations of QP–AL and QP–C Continuous Phases and o/w Emulsion Gels

The flow properties of the continuous phases and emulsions at 25 °C were determined using an AR-G2 rheometer (TA Instruments Ltd., Fleming Way, West Sussex, UK) with a cone-and-plate geometry (40 mm diameter; gap distance of 0.05 mm). The shear rate increased logarithmically from 0.1 to 100 s^−1^. Subsequently, the shear rate remained constant at 100 s^−1^ for 60 s and ultimately decreased logarithmically back to 0.1 s^−1^. The experimental values obtained for QP–AL were fitted to the Cross equation ([13]; the parameters η∞, η_0_, α and n were fitted to a multifactor non-linear regression (Statgraphics Centurion XV) as follows (Formula (1). Cross equation):

(1) $$\eta =\eta_{\infty }+\frac{\eta_{0}-\;\eta_{\infty }}{1+{\left(\alpha *\;\gamma \right)}^{n}}$$

where η_∞_ and η_0_ are the limiting viscosities at extremely low and extremely high shear rates, respectively. α is a characteristic time constant with the dimensions of time associated with the rupture of linkages in the structure of the fluid, and n is a dimensionless rate constant indicating the degree of dependence of viscosity on shear rate in the shear-thinning region [13].

The viscoelastic behaviour of the emulsion gels was determined by performing frequency sweeps on an AR-G2 rheometer (TA Instruments Ltd. Fleming Way, West Sussex, UK) at 25 °C using a cone-and-plate geometry with a 40-mm diameter. The storage or elastic modulus (G′), loss or viscous modulus (G″) and tan δ (G″/G′) were recorded over an oscillation frequency range of 0.1–10 Hz by applying a constant strain within the linear viscoelastic region of the emulsions (0.6% and 2% for QP–AL and QP–C, respectively).

### 4.5. Microstructural Characterisations of QP–AL and QP–C Emulsions

The droplet size distribution was determined by a Mastersizer Hydro 2000 MU microparticle analyser (Malvern Instruments, Malvern, UK) at a pump speed of 2000 rpm. The primary parameters obtained from the volumetric distribution were the polydispersity index (span), volumetric mean diameter (D [4, 3]) and Sauter mean diameter (D [3, 2]). The microstructure of the emulsion gels was determined by optical microscopy. One drop of each sample was examined at 40× magnification on an Axiostar Plus optical microscope (Carl Zeiss, Oberkochen, Germany) equipped with a PowerShot A570 IS digital camera (Canon, Melville, NY, USA) to capture images [27].

### 4.6. Estimation of the Creaming Rate of QP–C and QP–Al o/w Emulsions

Stokes’ law was used to estimate the creaming rate per the following equation [28] (Formula (2). Creaming rate):

(2) $$\upsilon_{s}=\frac{2a^{2}\left(\rho_{0}-\rho \right)g}{9\eta_{0}}$$

where a is the oil droplet radius estimated from D [4, 3] measured per the procedure described in Section 2.5; *g* is the acceleration due to gravity; ρ_0_ and ρ are the densities of the dispersed oil (sunflower oil) estimated from [29] and the continuous water phase, respectively; and η_0_ is the experimental viscosity at 0.1 s^−1^.

This creaming speed value, *ν*s, was corrected per the following semi-empirical equation, describing creaming in a moderately concentrated emulsion [28] (Formula (3). Adjusted creaming rate):

(3) $$\upsilon =\upsilon_{s}{\left[1-\frac{\emptyset }{\emptyset^{*}}\right]}^{k\emptyset^{*}}$$

where *ν*s is the Stokes creaming speed; *ϕ* (= 0.5) is the volume oil/water ratio; ϕ* is a parameter (≈0.6) approximating the close packing of monodisperse spheres; and k (≈0.8) is an adjustable parameter.

### 4.7. Determination of Emulsion Gel Stability Against Creaming

In total, 10 mL of fresh emulsions were transferred to 10 mL graduated tubes and stored in a refrigerator (5 °C). The total height of the emulsion gel and serum layer was used to quantify creaming. Changes in the height of the serum layer were measured regularly (on days 0, 1, 7, 14 and 28) [30]. The overall stability of the sample was evaluated using a Turbiscan Lab vertical scan analyser (Microtrac Formulaction, Toulouse, France) through backscattering measurements with a pulsed light source (λ = 850 nm) based on the height of the cylindrical glass tube containing the sample [31,32]. Light backscattering profiles of emulsion gels versus tube height were obtained based on storage time (on days 1, 7, 18, 25 and 29). The emulsions were stored at 5 °C and measured at 25 °C.

To determine the exact position of this interface at each time point, the backscattering data were fitted to the Boltzmann sigmoid equation over the height range 0–10,000 μm (Formula (4). Boltzmann sigmoid equation).

(4) $$BS\left(h\right)=BS_{bottom}+\frac{BS_{top}-BS_{bottom}}{1+e^{\frac{\left(h-H_{50}\right)}{dx}}}$$

where h is the height (μm); BS(h) is the backscattering % at that height; BS_bottom_ is the baseline backscattering (clear layer, approximately 4.5%); B_Stop_ is the maximum backscattering (cream layer, approximately 82%), and H50 is the height of the inflexion point. H50 represents the exact position of the creaming front; and dx is the width of the interface (sharpness). The H50 parameter was selected over the global Turbiscan Stability Index (TSI) because it provides a direct, physically quantifiable measure of the localized phase separation boundary migration. While TSI aggregates all structural changes across the entire tube height into a single dimensionless score, tracking H50 allows the precise calculation of the interface migration velocity (µ/day), which directly correlates with the physical creaming kinetics investigated in this work.

### 4.8. Optical Microscopy

The microstructure of the emulsions was determined by optical microscopy. One drop of each emulsion was examined at 40× magnification on an Axiostar Plus optical microscope (Carl Zeiss, Germany) equipped with a PowerShot A570 IS digital camera (Canon, USA) to capture the images [27].

### 4.9. Preparation and Characterisation of the Low-Fat Dressing

Various ingredients were added to the selected optimum emulsion gel to impart the organoleptic characteristics of a food dressing, such as a non-caloric flavouring, a non-caloric colourant and various natural spices to taste.

Based on the formula used to prepare this low-fat dressing, its nutritional composition per suggested portion (10 g) was determined, and its caloric content was calculated using the following formula [21,30,33,34] (Formula (5)—Estimated caloric value per suggested portion):

(5) $$Caloric\;value=\left(4\;Kcal\times g.protein\right)+\left(9\;Kcal\times g.fat\right)+(4\;Kcal\times g.carbohydrate)$$

A consumer acceptability test (hedonic) was used for the sensory characterisation of the low-fat dressing. A five-point hedonic scale (1 = I dislike it a lot, 2 = I dislike it, 3 = I neither like it nor dislike it, 4 = I like it and 5 = I truly like it) was used to evaluate various organoleptic attributes (appearance, aroma, colour, taste, texture and overall acceptability) of the dressing. For sensory evaluation, protocols were implemented to protect the rights and privacy of all participants throughout the research. All participants were informed of the ingredients used and provided their verbal informed consent before the sensory evaluation.

### 4.10. Statistical Analysis

All experimental results are presented as mean ± standard deviation. An analysis of variance (ANOVA), followed by Tukey’s multiple-range tests (*p* < 0.05), was performed using Statgraphics Centurion XV to determine significant differences between the different formulations.

## Figures and Tables

**Table 2:** Nutritional composition and caloric value of food dressing.

Nutrient	g/Portion (10 g)	kcal/Portion (10 g)
Carbohydrates	0.11	0.44
Proteins	0.10	0.40
Total fat	2.88	25.92
Saturated fat	0.31	2.79
Trans fat	0	0
Monounsaturated fat	0.70	6.3
Polyunsaturated fat	1.87	16.83
Cholesterol	0	0
Insoluble fibre	0.20	0
Total		26.76

## Data Availability

The data that support the findings of this study are available on request from the corresponding author.

## Supplementary Materials

The following supporting information can be downloaded at: https://www.mdpi.com/article/10.3390/gels12080699/s1, Figure S1: Creaming stability of emulsions: (A) Creaming index (%) as a function of storage time (days) for Alg and Qo formulations; visual creaming stability tubes for (B) QP–AL 1:4 and (C) QP–C 1:4 emulsions. Figure S2: Stability images of (A) QP–AL 1:4 and (B) QP–C 1:4 emulsions evaluated by light backscattering (Turbiscan). Figure S3: Visual appearance of (A) emulsion formulation components and (B) final food dressing application.

## Abbreviations

The following abbreviations are used in this manuscript:

Al	Sodium alginate/alginate
BS	Backscattering
C	Chitosan
D [3, 2]	Sauter mean diameter
D [4, 3]	Volumetric mean diameter
DA	Degree of acetylation
EQP–AL	Quinoa protein–alginate emulsion
EQP–AL8	Alginate-based emulsion containing 2% w/v of alginate
EQP–C	Quinoa protein–chitosan emulsion
EQP–C8	Chitosan-based emulsion containing 1.6% w/v of chitosan
G′	Storage or elastic modulus
G″	Loss or viscous modulus
H50	Height of the inflexion point/creaming front
M/G	Mannuronic acid/guluronic acid ratio
Mw	Average molar mass/molecular weight
o/w	Oil-in-water
pI	Isoelectric point
PDI	Polydispersity index/uniformity
QP	Quinoa protein
QP–AL/QP–Alg	Quinoa protein–alginate complexes
QP–C	Quinoa protein–chitosan complexes
QPI	Quinoa protein isolate
w/v	Weight/volume
XG	Xanthan gum

## References

[1] Aschemann-Witzel J., Gantriis R.F., Fraga P., Perez-Cueto F.J.A. (2021). Plant-based food and protein trend from a business perspective: Markets, consumers, and the role of innovation. Trends Food Sci. Technol..

[2] McClements D.J., Grossmann L. (2021). The science of plant-based foods: Constructing next-generation meat, dairy, and egg alternatives. Compr. Rev. Food Sci. Food Saf..

[3] Kyriakopoulou K., Dekkers B., van der Goot A.J. (2019). Plant-based meat analogues. Sustainable Meat Production and Processing.

[4] Abugoch L.E., Romero N., Tapia C.A., Silva J., Rivera M. (2008). Study of Some Physicochemical and Functional Properties of Quinoa (Chenopodium quinoa Willd) Protein Isolates. J. Agric. Food Chem..

[5] Qu G., Yang F., He X., Gao Y., Sun S. (2025). Quinoa protein-based emulsifiers: Mechanisms, influencing factors, modification techniques and applications. Food Chem..

[6] Ravindran S., Williams M.A.K., Ward R.L., Gillies G. (2018). Understanding how the properties of whey protein stabilized emulsions depend on pH, ionic strength and calcium concentration, by mapping environmental conditions to zeta potential. Food Hydrocoll..

[7] Chuah A.M., Kuroiwa T., Kobayashi I., Nakajima M. (2014). The influence of polysaccharide on the stability of protein stabilized oil-in-water emulsion prepared by microchannel emulsification technique. Colloids Surf. A Physicochem. Eng. Asp..

[8] Dickinson E. (2019). Strategies to control and inhibit the flocculation of protein-stabilized oil-in-water emulsions. Food Hydrocoll..

[9] Sriprablom J., Luangpituksa P., Wongkongkatep J., Pongtharangkul T., Suphantharika M. (2019). Influence of pH and ionic strength on the physical and rheological properties and stability of whey protein stabilized o/w emulsions containing xanthan gum. J. Food Eng..

[10] Romo I., Abugoch L., Tapia C. (2020). Soluble complexes between chenopodins and alginate/chitosan: Intermolecular interactions and structural-physicochemical properties. Carbohydr. Polym..

[11] Patole S., Cheng L., Yang Z. (2022). Impact of incorporations of various polysaccharides on rheological and microstructural characteristics of heat-induced quinoa protein isolate gels. Food Biophys..

[12] Calero N., Muñoz J., Ramírez P., Guerrero A. (2010). Flow behaviour, linear viscoelasticity and surface properties of chitosan aqueous solutions. Food Hydrocoll..

[13] Mancini M., Moresi M., Sappino F. (1996). Rheological behaviour of aqueous dispersions of algal sodium alginates. J. Food Eng..

[14] Romero A., Cordobés F., Puppo M.C., Guerrero A., Bengoechea C. (2008). Rheology and droplet size distribution of emulsions stabilized by crayfish flour. Food Hydrocoll..

[15] Worrasinchai S., Suphantharika M., Pinjai S., Jamnong P. (2006). β-Glucan prepared from spent brewer’s yeast as a fat replacer in mayonnaise. Food Hydrocoll..

[16] Laca A., Sáenz M.C., Paredes B., Díaz M. (2010). Rheological properties, stability and sensory evaluation of low-cholesterol mayonnaises prepared using egg yolk granules as emulsifying agent. J. Food Eng..

[17] Kontogiorgos V., Biliaderis C.G., Kiosseoglou V., Doxastakis G. (2004). Stability and rheology of egg-yolk-stabilized concentrated emulsions containing cereal β-glucans of varying molecular size. Food Hydrocoll..

[18] Brownlee I.A., Seal C.J., Wilcox M., Dettmar P.W., Pearson J.P. (2009). Applications of Alginates in Food.

[19] Day L., Zhai J., Xu M., Jones N.C., Hoffmann S.V., Wooster T.J. (2014). Conformational changes of globular proteins adsorbed at oil-in-water emulsion interfaces examined by Synchrotron Radiation Circular Dichroism. Food Hydrocoll..

[20] Downing D.L. (1996). Mayonnaise and Salad Dressing Products. A Complete Course in Canning and Related Processes.

[21] Gu J., Xin Z., Meng X., Sun S., Qiao Q., Deng H. (2016). A “reduced-pressure distillation” method to prepare zein-based fat analogue for application in mayonnaise formulation. J. Food Eng..

[22] Rashed A.A., Md Noh M.F., Mustafa Khalid N., Ab Rahman N.I., Tasirin A., Wan Omar W.S., Nawi M.N.M., Jamilan M.A., Selamat R. (2017). The nutritional composition of mayonnaise and salad dressing in the Malaysian market. Sains Malays..

[23] Wang Y., Zheng H., Li Y., Li B., Chen Y. (2015). One step procedure for desalting salty egg white and preparing fat analogue and its application in mayonnaise. Food Hydrocoll..

[24] Minsal (2019). Reglamento Sanitario De Los Alimentos.

[25] Gamboa A., Araujo V., Caro N., Gotteland M., Abugoch L., Tapia C. (2015). Spray Freeze-Drying as an Alternative to the Ionic Gelation Method to Produce Chitosan and Alginate Nano-Particles Targeted to the Colon. J. Pharm. Sci..

[26] Goulding D.A., Fox P.F., O’Mahony J.A., Boland M., Singh H. (2020). Milk proteins: An overview. Milk Proteins: From Expression to Food.

[27] Chung C., Degner B., McClements D.J. (2012). Rheology and microstructure of bimodal particulate dispersions: Model for foods containing fat droplets and starch granules. Food Res. Int..

[28] Dickinson E., Phillips G.O., Williams P.A. (2009). Hydrocolloids and emulsion stability. Handbook of Hydrocolloids.

[29] Esteban B., Riba J.R., Baquero G., Rius A., Puig R. (2012). Temperature dependence of density and viscosity of vegetable oils. Biomass Bioenergy.

[30] Shen R., Luo S., Dong J. (2011). Application of oat dextrine for fat substitute in mayonnaise. Food Chem..

[31] López-Castejón M.L., Bengoechea C., Espinosa S., Carrera C. (2019). Characterization of prebiotic emulsions stabilized by inulin and β-lactoglobulin. Food Hydrocoll..

[32] Mengual O., Meunier G., Cayré I., Puech K., Snabre P. (1999). TURBISCAN MA 2000: Multiple light scattering measurement for concentrated emulsion and suspension instability analysis. Talanta.

[33] Li J., Wang Y., Jin W., Zhou B., Li B. (2014). Application of micronized konjac gel for fat analogue in mayonnaise. Food Hydrocoll..

[34] Liu H., Xu X.M., Guo S.D. (2007). Rheological, texture and sensory properties of low-fat mayonnaise with different fat mimetics. LWT-Food Sci. Technol..

