# Supplementary material for: Soluble Chenopodin–Alginate and Chenopodin–Chitosan Nanocomplexes as Building Blocks for Food Emulsion Gels
Source: Gels. 2026 Aug 5;12(8):699. doi: 10.3390/gels12080699 (PMC13512242; doi:10.3390/gels12080699)
Supplement: Supplementary file 1 [file gels-12-00699-s001.zip › gels-4410465-supplementary.pdf]

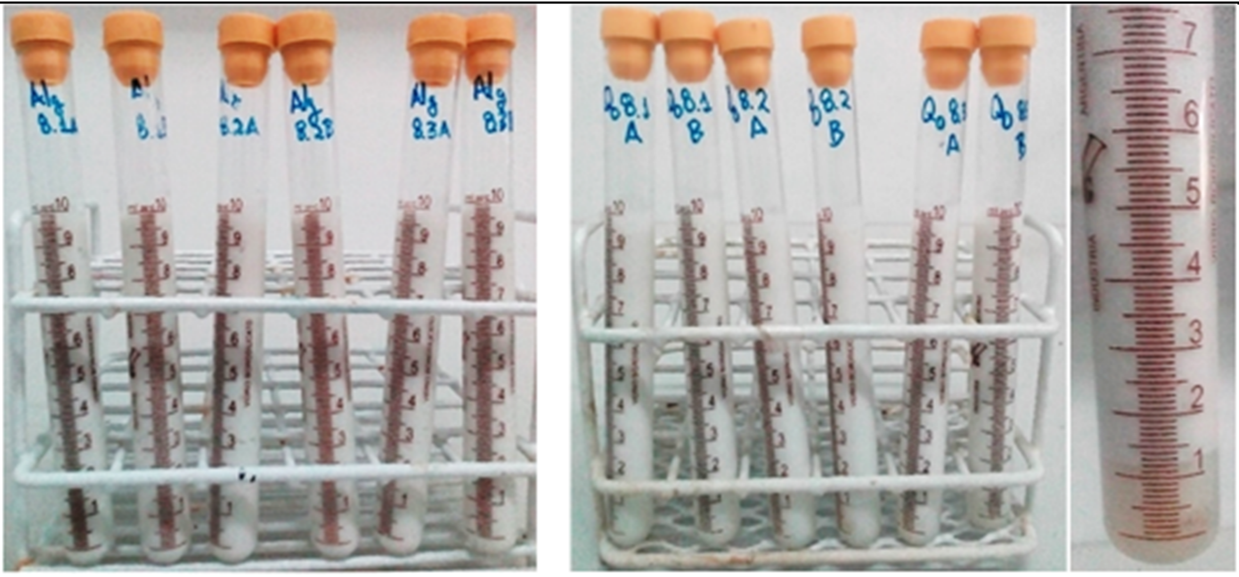

(A)	(B)	(C)

Figure S1. Creaming stability of emulsions: (A) Creaming index (%) as a function of storage time (days) for Alg and Qo formulations; visual creaming stability tubes for (B) QP–AL 1:4 and (C) QP–C 1:4 emulsions.

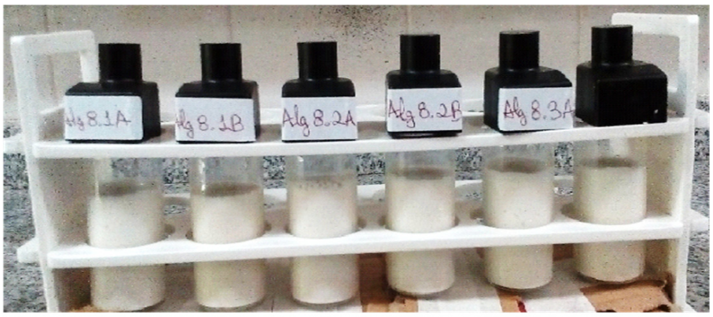

(A)

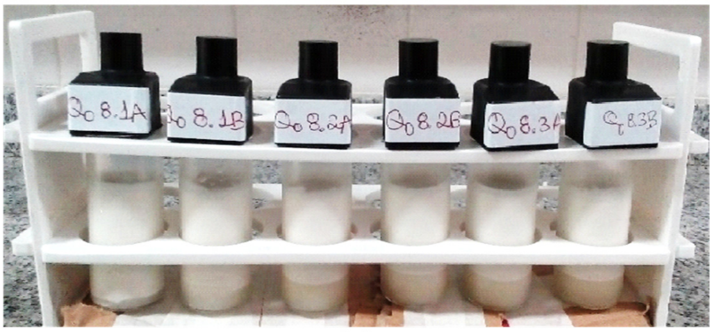

(B)

Figure S2. Stability images of (A) QP–AL 1:4 and (B) QP–C 1:4 emulsions evaluated by light. backscattering (Turbiscan).

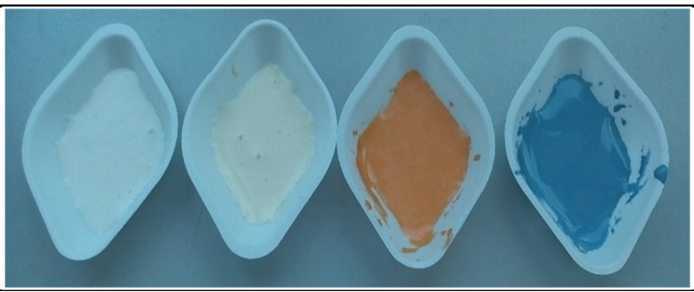

(A)

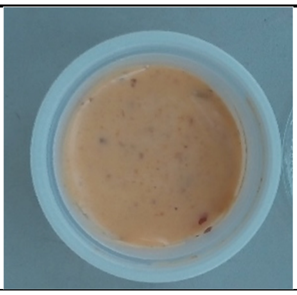

(B)

Figure S3. Visual appearance of (A) emulsion formulation components and (B) final food dressing application.
